# Vitamin D both facilitates and attenuates the cellular response to lipopolysaccharide

**DOI:** 10.1038/srep45172

**Published:** 2017-03-27

**Authors:** Ling Chen, Mathew Suji Eapen, Graeme R. Zosky

**Affiliations:** 1 School of Medicine, Faculty of Health, University of Tasmania, Hobart, Tasmania, 7000, Australia

## Abstract

Vitamin D has a range of non-skeletal health effects and has been implicated in the response to respiratory infections. The aim of this study was to assess the effect of vitamin D on the response of epithelial cells, neutrophils and macrophages to lipopolysaccharide (LPS) stimulation. BEAS-2B cells (airway epithelial cell line) and primary neutrophils and macrophages isolated from blood samples were cultured and exposed to LPS with and without vitamin D (1,25(OH)_2_D). The production of IL-6, IL-8, IL-1β and TNF-α of all cells and the phagocytic capacity of neutrophils and macrophages to *E. coli* were assessed. Vitamin D had no effect on BEAS-2B cells but enhanced the production of IL-8 in neutrophils (p = 0.007) and IL-1β in macrophages (p = 0.007) in response to LPS. Both vitamin D (p = 0.019) and LPS (p < 0.001) reduced the phagocytic capacity of macrophages. These data suggest that the impact of vitamin D on responses to infection are complex and that the net effect will depend on the cells that respond, the key response that is necessary for resolution of infection (cytokine production or phagocytosis) and whether there is pre-existing inflammation.

Vitamin D is a secosteroid hormone which is well-known of its role in mineral and skeletal homeostasis^1^. A plethora of studies have suggested that vitamin D has a range of roles beyond the regulation of bone metabolism and that it plays a critical role in modulating the immune response; including the response to respiratory infections^2^. Respiratory tract infections (RTI), which are highly prevalent, are responsible for significant morbidity and mortality, and are associated with the onset, progression and exacerbation of chronic lung diseases^3^,^4^.

Epidemiological studies have demonstrated strong associations between serum vitamin D levels and the incidence of RTIs^5^ and it has been suggested that the seasonal variations in vitamin D levels could explain the increased prevalence of RTIs in winter^6^, when vitamin D synthesis is low^7^. However, clinical trials with vitamin D supplementation have reported varying effects of on responses to respiratory infections^8^,^9^ such that current evidence linking vitamin D status and RTIs is equivocal.

Airway epithelial cells, as the first defensive barrier in the airway tract, play an important role in orchestrating neutrophil and macrophage recruitment to clear invading pathogens^10^. Neutrophils and macrophages play an important role in clearing pathogens through their phagocytic capacity by producing oxidants to kill engulfed microorganisms^11^. It is believed that the local production of 1α, 25-Dihydroxyvitamin D_3_ (1,25(OH)_2_D_3_), the active form of vitamin D, exerts protective effects during infections by upregulating the expression of cathelicidin and β defensin 2 in phagocytes^12^,^13^ and epithelial cells^14^. The clearance of pathogens and apoptotic cells by phagocytosis plays an important role in resolving inflammatory responses, and any impairment of these processes can lead to a chronic inflammatory state^15^.

While vitamin D is thought to be important in mediating the immune response to infection, as discussed above, the role of vitamin D in modulating cytokines production is unclear. Previous studies have shown conflicting effects (anti-inflammatory and pro-inflammatory) of vitamin D on the production of IL (interleukin) -1β, IL-6 and TNF (tumor necrosis factor) -α in response to a variety of insults^16^,^17^,^18^. IL-1β and TNF-α are essential for the activation of the epithelium and downstream inflammatory responses^19^. In addition, IL-1β, IL-6, IL-8 and TNF-α are involved in the innate immune responses by neutrophils and macrophages^20^ and may be influenced by vitamin D^16^,^17^,^18^,^21^.

Given the inconsistencies in the *in vitro* and clinical trial data, we aimed to determine the effect of exogenous vitamin D on the inflammatory response in key cells involved in the response to RTIs including epithelial cells, macrophages and neutrophils. In order to achieve this, we examined whether vitamin D modulated the response to bacterial lipopolysaccharide (LPS).

## Results

### Cytokine production by BEAS-2B cells

**Figure 1:**
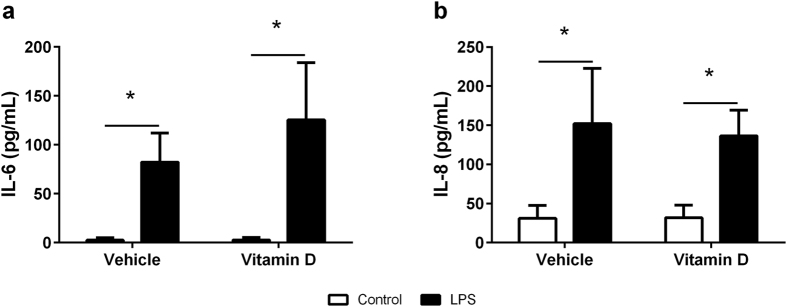
Production of IL-6 (**a**) and IL-8 (**b**) in the supernatant of BEAS-2B cells with (black bars) and without (white bars) LPS in the presence of vehicle or vitamin D (1,25(OH)_2_D_3_). Data are represented as mean (SD), *indicates p < 0.05, n = 4/group.

24 hours after exposure to LPS, the production of IL-6 (Fig. 1a, p < 0.001) and IL-8 (Fig. 1b, p < 0.001) was increased in BEAS-2B cells compared to controls. However, the magnitude of the response was not altered by the presence of 1,25(OH)_2_D_3_ (IL-6, p = 0.552; IL-8, P = 0.994). IL-1β and TNF-α were not detectable by ELISA in any of the supernatants (*data not shown*).

### Cytokine production by neutrophils

**Figure 2:**
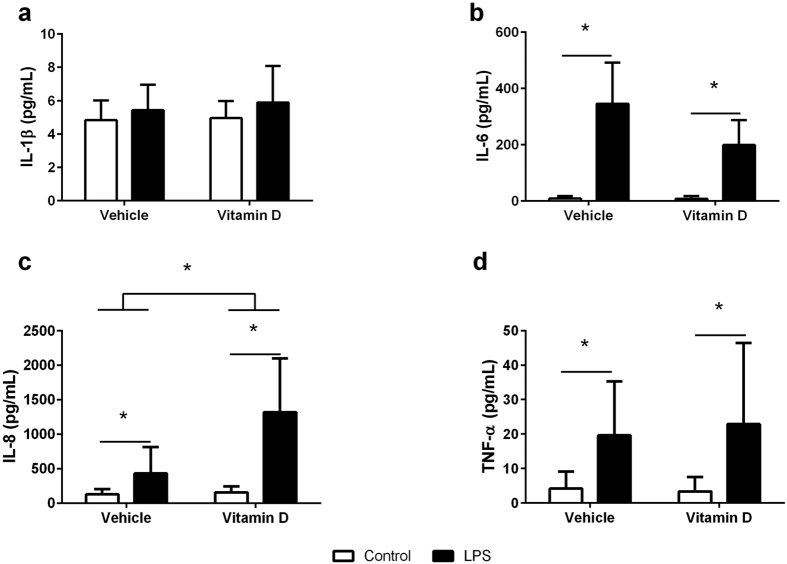
Production of IL-1β (**a**), IL-6 (**b**), IL-8 (**c**) and TNF-α (**d**) in the supernatant of neutrophils with (black bars) and without (white bars) LPS in the presence of vehicle or vitamin D (1,25(OH)_2_D_3_). Data are represented as mean (SD), *indicates p < 0.05, n = 9/group for IL-1β testing, n = 5/group for IL-6 testing, n = 8/group for IL-8 testing, and n = 9/group for TNF-α testing.

24 hours after exposure to LPS, the production of IL-6 (Fig. 2b, p < 0.001), IL-8 (Fig. 2c, p < 0.001) and TNF-α (Fig. 2d, p = 0.002) was increased in neutrophils compared to controls. Interestingly, while 1,25(OH)_2_D_3_ had no effect on the production of IL-6 (p = 0.297) or TNF- α (p = 0.728) by the neutrophils, it increased the IL-8 response (p = 0.007) in both the LPS treated and untreated neutrophils. Neither LPS (p = 0.153) nor 1,25(OH)_2_D_3_ had any effect on IL-1β production (Fig. 2a, p = 0.600).

### Cytokine production by macrophages

**Figure 3:**
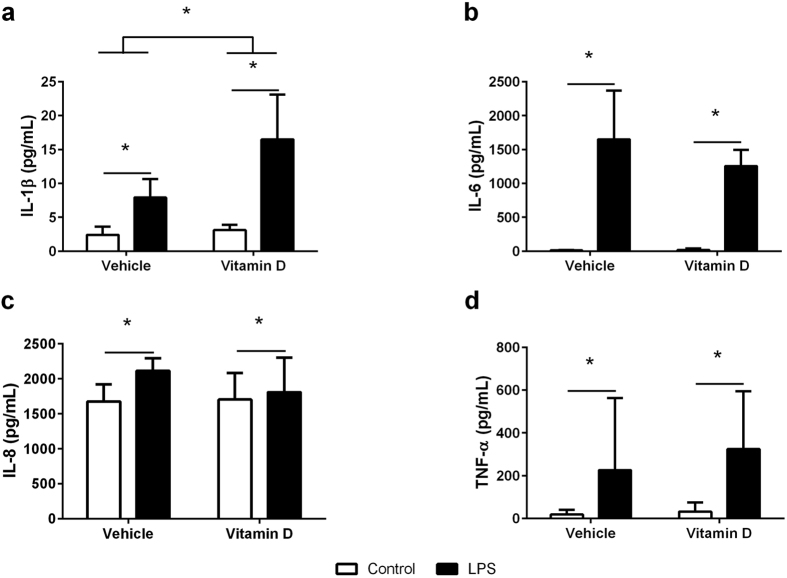
Production of IL-1β (**a**), IL-6 (**b**), IL-8 (**c**) and TNF-α (**d**) in the supernatant of macrophages with (black bars) and without (white bars) LPS in the presence of vehicle or vitamin D (1,25(OH)_2_D_3_). Data are represented as mean (SD), *indicates p < 0.05, n = 7/group for IL-1β testing, n = 5/group for IL-6 testing, n = 7/group for IL-8 testing, and n = 8/group for TNF-α testing.

24 hours after exposure to LPS, the production of IL-1β (Fig. 3a, p < 0.001), IL-6 (Fig. 3b, p < 0.001), IL-8 (Fig. 3c, p = 0.049) and TNF-α (Fig. 3d, p < 0.001) was increased in macrophages compared to controls. In contrast to the neutrophils, while 1,25(OH)_2_D_3_ had no effect on the production of IL-6 (p = 0.531), IL-8 (p = 0.297) or TNF-α (p = 0.095), it increased the production of IL-1β (p = 0.001) in both the LPS treated and untreated cells.

### Phagocytic capacity

**Figure 4:**
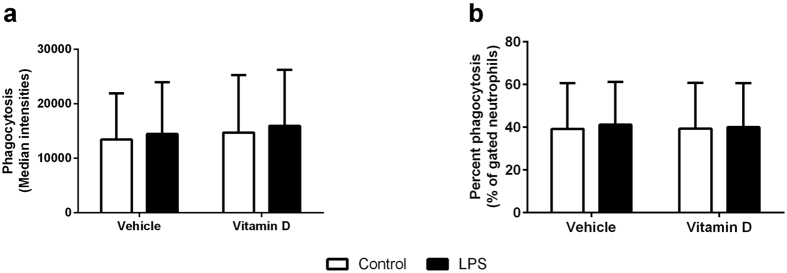
Phagocytic capacity of neutrophils measured as the total phagocytosis of *E. coli* (**a**) or the percentage of cells that phagocytosed *E. coli* (**b**) in neutrophils treated with (black bars) or without (white bars) LPS in the presence of vehicle or vitamin D (1,25(OH)_2_D_3_). Data are represented as mean (SD), n = 6/group.

**Figure 5:**
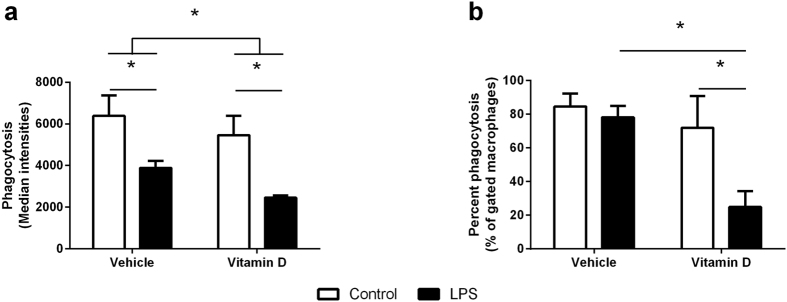
Phagocytic capacity of macrophages measured as the total phagocytosis of *E. coli* (**a**) or the percentage of cells that phagocytosed *E. coli* (**b**) in macrophages treated with (black bars) or without (white bars) LPS in the presence of vehicle or vitamin D (1,25(OH)_2_D_3_). Data are represented as mean (SD), *indicates p < 0.05, n = 3/group.

There was no effect of LPS treatment or 1,25(OH)_2_D_3_ on total phagocytic capacity (Fig. 4a) (LPS, p = 0.732; 1,25(OH)_2_D_3_, p = 0.802) or the percentage of cells that engulfed the *E. coli* (Fig. 4b) (LPS, p = 0.788; 1,25(OH)_2_D_3_, p = 0.936) in neutrophils. In contrast, both LPS (p < 0.001) and 1,25(OH)_2_D_3_ (p = 0.019) reduced the phagocytic capacity of macrophages (Fig. 5a). In the case of LPS and 1,25(OH)_2_D_3_, in isolation, the effect seemed to be due to the reduced capacity of individual cells because the percentage of cells that phagocytosed *E. coli* was not changed in response to LPS (p = 0.516) or 1,25(OH)_2_D_3_ (p = 0.219) alone (Fig. 5b). However, in combination, LPS and 1,25(OH)_2_D_3_ significantly reduced the number of macrophages with phagocytic capacity (Fig. 5b, p < 0.001).

## Discussion

Data on the effect of vitamin D deficiency on responses to respiratory tract infections are conflicting. We aimed to examine the effect of supplemental vitamin D on cytokine production and phagocytic capacity in three cell types that are important in the innate response to respiratory infections; airway epithelial cells, neutrophils and macrophages. We found that 1,25(OH)_2_D_3_, the active form of vitamin D, enhanced IL-8 and IL-1β production by neutrophils and macrophages respectively. 1,25(OH)_2_D_3_ also decreased the total phagocytic potential of macrophages and, in combination with LPS, reduced the ability of individual macrophages to engulf *E. coli*. These data suggest that vitamin D has differing effects on the response to individual cell types to a bacterial stimulus. On the one hand the addition of vitamin D enhanced the production of cytokines but reduced the capacity of the macrophages to phagocytose pathogens. This may explain the conflicting associations between vitamin D and respiratory tract infections.

The airway epithelium is the first point of contact for invading pathogens in the respiratory tract. In response to bacterial stimuli, airway epithelial cells produce a range of pro-inflammatory mediators such as IL-6 and IL-8^22^ which act to recruit mononuclear phagocytes^23^ and neutrophils^24^. These phagocytes play important roles in responding to, and clearing respiratory infections by phagocytosing and producing reactive oxygen species (ROS) to eliminate pathogens^25^. As expected, we saw increased production of IL-6 and IL-8 by airway epithelial, BEAS-2B, cells in response to LPS stimulation. These responses were not altered by vitamin D suggesting that vitamin D does not modulate important innate responses by epithelial cells to bacterial infections in the airway.

Macrophages and neutrophils are key cells in generating the innate immune response to invading pathogens. When a pathogen crosses the epithelial barrier and begins to replicate in the tissues of the host, it is immediately recognized by macrophages, that reside in the tissues, which, along with epithelial cells, produce chemokines to recruit large numbers of neutrophils to sites of infection^10^. Macrophages and neutrophils respond to pathogens, in part, by releasing a plethora of cytokines and chemokines in order to orchestrate the immune response^26^. Neutrophils have a high phagocytic capacity and, when a pathogen is engulfed, release IL-8, a neutrophil chemoattractant^27^, which recruits additional neutrophils to site of infection^24^. Neutrophils also act to eliminate the pathogen by producing an oxidative burst^28^. As expected, we found that LPS increased the production of IL-8 by neutrophils. Interestingly, 25(OH)_2_D_3_ in our study facilitated the production of IL-8 suggesting that vitamin D may boost the capacity of neutrophils to respond to invading pathogens by recruiting additional neutrophils to the site of infection. This is in contrast to other studies on respiratory pathogens including tuberculosis where vitamin D had no effect on neutrophilia^29^. Similarly, vitamin D has been shown down-regulate IL-8 release in hyper-inflammatory macrophages^21^. The disparities in these observations suggest that the impact of vitamin D on the production of IL-8 by innate immune cells is dependent on the cell that is producing the cytokine and the infectious stimulus.

Activated macrophages produce a range of inflammatory mediators in response to bacterial infections. Consistent with previous studies^30^, we found that macrophages increased production of all the cytokines measured, IL-1β, IL-6, IL-8 and TNF-α, in response to LPS stimulation. However, pre-treatment with 1,25(OH)_2_D_3_ during macrophage differentiation period facilitated LPS induced IL-1β production but had no effects on the production of IL-6, IL-8 and TNF-α which is in contrast to a recent study which showed that 1,25(OH)_2_D_3_ significantly reduced the levels of IL-6 and TNF-α in alveolar macrophages in response to a combination of LPS and IFN-γ^31^. It is important to note that our study used differentiated PBMCs that were primed with vitamin D during differentiation rather than primary alveolar macrophage, so it is possible that the inconsistencies in these studies reflects different macrophage phenotypes. Again, this suggest that vitamin D can both potentiate and inhibit the inflammatory response under different conditions.

Our study is also in contrast to an early study which suggested that 1,25(OH)_2_D_3_ inhibits the proliferation of blood lymphocytes and IL-1 production^32^, however, recent studies, in line with our data, have found that vitamin D generally boosts infection-stimulated cytokine and chemokine responses; including enhanced IL-1β expression in macrophages^18^, enhanced macrophage survival and reduced mycobacterial burden by stimulating the anti-mycobacterial capacity of co-cultured lung epithelial cells. IL-1β exerts its protective action against infections by rapidly recruiting neutrophils to inflammatory sites. For example, IL-1 receptor knock-out mice recruit fewer neutrophils in response to influenza infection^33^. In addition, *in vitro* studies have shown that IL-1β restricts intracellular *Mycobacterium tuberculosis (M. tuberculosis*) growth in murine and human macrophages by regulating TNF signalling and caspase-3 activation^34^. Similar to our observation in neutrophils, these data suggested that vitamin D enhances the capacity of macrophages to respond to bacterial stimuli.

Phagocytosis of pathogens by neutrophils and macrophages is one of their key functions^35^. While localization of neutrophils to the site of inflammation is crucial for clearance of the infection, which vitamin D appears to enhance through increased IL-8 production, neither LPS nor vitamin D had any impact on the phagocytic capacity of neutrophils when challenged with *E. coli*. Interestingly, a previous study has clearly demonstrated that vitamin D enhances the phagocytic potential of macrophages that have low phagocytic capacity to live *M. tuberculosis*^36^. In contrast, our results have shown that both LPS and 1,25(OH)_2_D_3_ independently decreased the phagocytic capacity of individual macrophages to engulf *E. coli* with a decrease in overall phagocytosis (overall fluorescence) but approximately the same number of cells involved in phagocytosis. Interestingly, LPS and 1,25(OH)_2_D_3_ together decreased the number of macrophages that were able to phagocytose *E. coli*. While there are several differences between these studies that may explain this difference, including the length of prior treatment with vitamin D (2 vs 6 days), the pathogen used, and the length of pathogen challenge (45 minutes vs 3 hours), these observation suggest that vitamin D is a potent modulator of the phagocytic capacity of macrophages. In line with our data, *in vivo* studies have shown that mice injected with LPS had reduced phagocytic capacity in macrophages that may be due to the fact that the LPS drives macrophages to a more inflammatory, antigen presentation state^37^. There are numerous receptors involved in bacterial recognition by macrophages^38^, such that, if the macrophage receptors involved in phagocytosis are reduced, occupied, or internalized by stimuli, the phagocytic capacity can be attenuated^39^.

Other studies have shown that administration of vitamin D (10^−8^ M of 1,25(OH)_2_D_3_) had no effects on the phagocytic capacity of human alveolar macrophages, but increased the levels of the antimicrobial peptide cathelicidin (LL-37)^31^. We assessed LL-37 levels in the supernatants from our BEAS-2B cell and macrophage experiments and found that LL-37 levels were not increased in macrophages by pre-stimulation with vitamin D (10^−7^ M of 1,25(OH)_2_D_3_) during differentiation and were undetectable in BEAS-2B cells (Supplementary material; Figure S1). This again highlights the importance of cell phenotype, and protocol, in influencing the cellular response to stimulation with vitamin D. Studies have shown that vitamin D has an effect on the morphology and function of monocyte-derived macrophages^40^ by inhibiting M1 macrophage activation and promoting M1-M2 phenotype switching^41^,^42^. However, our data appear to contradict this and it possible that by priming with vitamin D during differentiation we facilitated M1 phenotype switching with increased the production of IL-1β. These data have important implications for the clearance of pathogens whereby the effect of vitamin D on macrophage phagocytosis and antimicrobial activity seems to be dependent on the type of pathogen, the period of exposure to vitamin D, the presence of pre-existing inflammation, and macrophage phenotype.

In conclusion, vitamin D exerted different effects on different cell types. While there was no effect of vitamin D on LPS induced inflammation in epithelial cells, vitamin D enhanced the production of IL-8 in neutrophils and the production of IL-1β macrophages. Vitamin D had no effect on neutrophils phagocytic capacity but exaggerated the LPS induced decrease in macrophage phagocytic capacity. While acknowledging that we have studied these responses in isolation *in vitro*, and that these cells have the capacity to modulate 1,25(OH)_2_D levels *in vivo* through the upregulation of 25-hydroxyvitamin D-1α-hydroxylase (CYP27B1)^43^, our data demonstrate the complexity in understanding the effect of vitamin D on responses to infection. In some instances, the net effect of vitamin D on the response to infection may be neutral due to the competing effect of vitamin D on phagocytosis and the production of cytokines that are important in the resolution of infection. This area requires further investigation if we are to understand the complex role of vitamin D in the innate immune response.

## Methods

### Study subjects

Bronchial epithelial cells (BEAS-2B) were purchased from American Type Culture Collection (ATCC; Manassas, VA). Whole blood samples were collected from normal healthy (no acute or chronic illnesses) volunteers (aged 20–35 years; 5 males and 5 females) to isolate peripheral blood neutrophils and monocytes. The University of Tasmania Research Ethics Committee approved all the studies, and subjects gave written, informed consent. All methods were performed in accordance with the relevant guidelines and regulations.

### Neutrophil and monocyte isolation

Neutrophils and human peripheral blood mononuclear cells (PBMCs) were isolated by density gradient centrifugation using Lympholyte-poly cell separation media (Cedarlane Labs, Burlington, Canada) composed of sodium diatrizoate and dextran 500^44^. 30 mL of freshly drawn blood was collected in Vacutainer collection tubes (BD, San Jose, CA) containing 15% K_3_EDTA solution and then mixed at a ratio of 1:1 (vol/vol) with PBS at room temperature and 15 mL of the mixture was layered with 15 mL of Lympholyte-poly in a 50 mL tube. After centrifugation at 450 g for 35 min at 20 °C, neutrophils and PBMCs buffy coats were collected separately. Neutrophils buffy coats were washed with ice-cold HBSS (without Ca^2+^/Mg^2+^) and centrifuged at 450 g for 5 min. The remaining red blood cells were lysed by hypotonic treatment with Red Blood Cell Lysis Buffer (Roche, Basel, Switzerland). This method has been show to yield samples of >95% neutrophils with >95% viability^45^. PBMCs buffy coats were washed with ice-cold PBS containing 0.1% BSA and 2 mM EDTA and centrifuged at 450 g for 5 min. Monocytes were purified from freshly isolated PBMCs using MACS CD14 microbeads according to the manufacturer’s introductions (Miltenyi Biotec, Bergisch Gladbach, Germany), which are used for positive selection of human monocytes and macrophages from PBMCs^46^. Briefly, monocytes suspension was passed through the Pre-Separation filter then incubated with CD14 microbeads for 15 min at 4 °C, to allow separation of CD14^+^ monocytes.

### Cytokines

The concentrations of interleukin-1 beta (IL-1β), interleukin-6 (IL-6), interleukin-8 (IL-8), and tumor necrosis factor alpha (TNF-α) in the supernatants from the cell culture medium were measured using DuoSet ELISA kits (R&D Systems, Minneapolis, MN). The absorbance was read at 450 nm/570nm using a spectrophotometer (Spectramax M2, Molecular Devices, Sunnyvale, CA).

### BEAS-2B inflammatory response

BEAS-2B cells were cultured in bronchial epithelial cell growth medium (BEGM) (Lonza, Walkersville, MD) at 37 °C in a 5% CO_2_ humidified incubator. BEAS-2B cells were seeded in 12-well plates at a density of 5 × 10^5^ cells/mL with either 0.1% ethanol (vehicle control) or 10^−7^ M of 1,25(OH)_2_D_3_ (Enzo Life Sciences, Farmingdale, NY) for 24 hours. After 24 hours, the culture media was changed, and cells were treated with 10 ng/mL of LPS (Sigma-Aldrich, St.Louis, MO) in the presence of vehicle control or 10^−7^ M of 1,25(OH)_2_D_3_ for 24 hours at which point cell supernatants were collected for enzyme-linked immune sorbent assay (ELISA) assessment of IL-1β, IL-6, IL-8 and TNF-α.

### Neutrophil inflammatory response

Isolated neutrophils were cultured in RPMI-1640 medium (Thermo Fisher Scientific, Waltham, MA) supplied with 10% fetal bovine serum (FBS) (Sigma-Aldrich, St.Louis, MO) and 1% Penicillin-Streptomycin (Pen-Strep) (Sigma-Aldrich, St.Louis, MO) at 37 °C in a 5% CO_2_ humidified incubator. Neutrophils were seeded in 12-well plates at a density of 1 × 10^6^ cells/mL with either 0.1% ethanol (vehicle control) or 10^−7^ M of 1,25(OH)_2_D_3_ for 24 hours prior to treatment with 10 ng/mL of LPS in the presence of vehicle control or 10^−7^ M of 1,25(OH)_2_D_3_ for another 24 hours. Cells suspensions were centrifuged and the supernatant collected for ELISA assessment of IL-1β, IL-6, IL-8 and TNF-α.

### Monocyte inflammatory response

The enriched CD14^+^ monocytes from PBMCs were cultured in RPMI-1640 medium supplied with 10% FBS and 1% Pen-Strep at 37 °C in a 5% CO_2_ humidified incubator. Monocytes were seeded in 12-well plates at a density of 5 × 10^5^ cells/mL, and differentiated using 100 ng/mL of macrophage colony stimulating factor (M-CSF) (Sigma-Aldrich, St.Louis, MO) in the presence of vehicle control or 10^−7^ M of 1,25(OH)_2_D_3_ for 6 days with fresh 1,25(OH)_2_D_3_ added every day^30^. At day 7, M-CSF containing media was changed, and PBMCs differentiated macrophages were treated with 10 ng/mL of LPS in the presence of vehicle control or 10^−7^ M of 1,25(OH)_2_D_3_ for 24 hours. The supernatants were centrifuged and the supernatant collected for ELISA assessment of IL-1β, IL-6, IL-8 and TNF-α.

### Phagocytic capacity of neutrophils and macrophages

Fluorescein conjugated Escherichia coli (K-12 strain) (Thermo Fisher Scientific, Waltham, MA) was used to assess phagocytic capacity. Briefly, after 24 hours of stimulation with LPS, neutrophils or macrophages were incubated with Fluorescein conjugated *E. coli* at multiplicity of infection (MOI) of 5 for 45 minutes at 37 °C in a 5% CO_2_ humidified incubator. The reaction was stopped with ice. Cells were collected and washed 3 times with cold PBS, then phagocytes were fixed in 1% paraformaldehyde before been measured by flow cytometer BD FACSCANTO II (BD Biosciences, San Jose, CA). Optimal removal of macrophages required vigorous pipetting. Flow cytometry was performed to estimate the percentage of phagocytes which had engulfed bacteria and quantify the fluorescence intensities of *E. coli* in engulfed phagocytes. Flow cytometry data were analysed using FCS Express 6 (De Novo Software, Glendale, CA).

### Statistical analysis

Group comparisons were made using two-way analysis of variance (Two-way ANOVA) with Holm-Sidak posthoc tests. Data were log-transformed where necessary to satisfy the assumptions of the test (normality and homoscedasticity). Data were analysed in SigmaPlot (Systat, Erkrath, Germany) and reported as mean (SD). P values of <0.05 were considered statistically significant.

## Additional Information

**How to cite this article:** Chen, L. *et al*. Vitamin D both facilitates and attenuates the cellular response to lipopolysaccharide. *Sci. Rep.* **7**, 45172; doi: 10.1038/srep45172 (2017).

**Publisher's note:** Springer Nature remains neutral with regard to jurisdictional claims in published maps and institutional affiliations.

## Supplementary Material

Supplementary Information
