# Supplementary material for: Vitamin D both facilitates and attenuates the cellular response to lipopolysaccharide
Source: Sci Rep. 2017 Mar 27;7:45172. doi: 10.1038/srep45172 (PMC5366921; doi:10.1038/srep45172)
Supplement: Supplementary Information [file srep45172-s1.pdf]

Supplementary material

Vitamin D both facilitates and attenuates the cellular response to lipopolysaccharide

Ling Chen[1], Mathew Eapen[1], Graeme R. Zosky[1]

[1]School of Medicine, Faculty of Health, University of Tasmania, Hobart, Tasmania, Australia, 7000

Results

LL-37 production by BEAS-2B cells and macrophages

24 hours after exposure to LPS, the production of LL-37 was not detectable by ELISA in the supernatants from BEAS-2B cells (*data not shown*). There was of effect of LPS treatment ($p = 0.06$) or $1,25(OH)_2D_3$ ($p = 0.09$) on the secretion of LL-37 from macrophages (Figure S1).

Methods

Cathelicidin (LL-37) production by BEAS-2B cells and monocytes

The supernatants from BEAS-2B cells and macrophages were collected for the assessment of levels of cathelicidin using the human LL-37 ELISA kit (Hycult Biotech, Plymouth Meeting, PA), according to the manufacturer's instruction.

Figures and figure legends

Figure S1

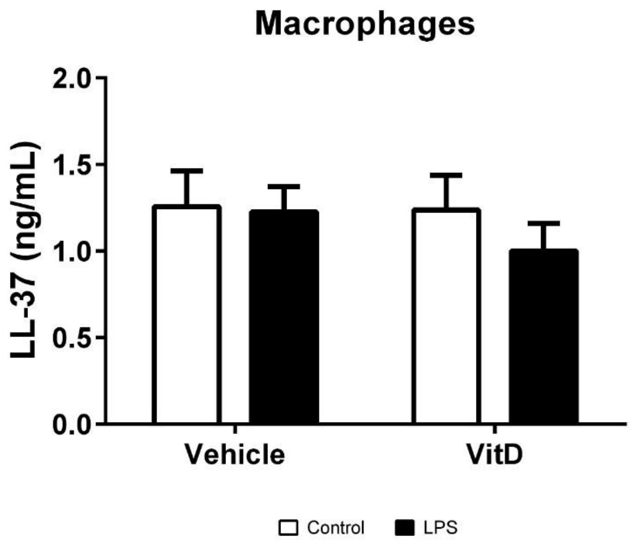

Figure S1: Production of LL-37 in the supernatant of macrophages with (black bars) and without (white bars) LPS in the presence of vehicle or vitamin D (1,25$(OH)_2D_3$). Data are represented as mean (SD), n = 7/group.
